# Preconception Partner-Specific Vitamin D Biomarkers and Risk of Preeclampsia Among Couples Undergoing Fertility Treatment: A Secondary Cohort Analysis of FAZST

**DOI:** 10.3390/nu18162597

**Published:** 2026-08-08

**Authors:** Zeina M. Alkhalaf, Neil J. Perkins

**Affiliations:** 1 Department of Family Science, School of Public Health, University of Maryland, College Park, MD 20742, USA; 2 Biostatistics Branch, Division of Intramural Population Health Research, Eunice Kennedy Shriver National Institute of Child Health and Human Development, National Institutes of Health, Bethesda, MD 20892, USA

**Keywords:** vitamin D, preeclampsia, preconception health, paternal health, free 25(OH)D, bioavailable 25(OH)D, placentation, assisted reproductive technology

## Abstract

Background/Objectives: Maternal vitamin D deficiency has been associated with preeclampsia, but evidence regarding preconception partner-specific vitamin D biomarkers is limited. We evaluated associations between preconception maternal and paternal vitamin D biomarkers and the odds of preeclampsia. Methods: This secondary prospective cohort analysis used data from the Folic Acid and Zinc Supplementation Trial (FAZST), a multicenter study conducted in the United States between 2013 and 2017. Of the 2370 enrolled couples who were seeking infertility treatment, 1054 couples achieved a documented pregnancy. Because preeclampsia can only be ascertained among pregnancies progressing beyond 20 weeks of gestation, the primary analytic population was restricted to 826 pregnancies that reached ≥20 weeks, including 98 with preeclampsia and 728 without preeclampsia. Preconception serum total 25-hydroxyvitamin D (25(OH)D), vitamin D-binding globulin (VDBG), albumin, free 25(OH)D, and bioavailable 25(OH)D were assessed in both partners. Multivariable logistic regression models estimated adjusted odds ratios (aORs) and 95% confidence intervals (CIs). Results: In categorical analyses, maternal total 25(OH)D findings were consistent with a possible U-shaped pattern. Compared with concentrations of 30–< 50 ng/mL, concentrations ≤20 ng/mL were associated with higher odds of preeclampsia (aOR = 2.58, 95% CI: 1.13–5.88), as were concentrations ≥50 ng/mL (aOR = 5.45, 95% CI: 1.71–17.41). Similar categorical patterns were observed for maternal free 25(OH)D, with higher odds at concentrations ≤8.25 pg/mL (aOR = 2.23, 95% CI: 1.13–4.43) and ≥12.5 pg/mL (aOR = 4.71, 95% CI: 1.97–11.29), and for maternal bioavailable 25(OH)D concentrations ≤3.5 ng/mL (aOR = 2.61, 95% CI: 1.19–5.72) and ≥5.0 ng/mL (aOR = 5.38, 95% CI: 2.15–13.46). Lower paternal bioavailable 25(OH)D was associated with lower odds of preeclampsia in the fully adjusted model (aOR = 0.50, 95% CI: 0.28–0.89); however, this finding should be considered exploratory. Conclusions: Among pregnancies progressing to ≥20 weeks, lower and higher categories of maternal total, free, and bioavailable 25(OH)D were associated with the odds of preeclampsia relative to intermediate categories. These categorical findings are consistent with a possible U-shaped pattern but do not establish a nonlinear dose–response relationship. Paternal findings were limited and should be considered exploratory.

## 1. Introduction

Preeclampsia is a hypertensive disorder that develops after 20 weeks of gestation and remains a significant cause of maternal and fetal morbidity and mortality worldwide [1,2]. Despite extensive research on its etiology, the pathophysiology of preeclampsia still remains unclear [3,4,5]. Increasing evidence suggests that the origins of preeclampsia may begin during implantation and placentation, with inflammatory and immunologic pathways playing a central role [6,7,8]. Paternal factors, including sperm-derived genetic and epigenetic signals, may also influence implantation and placental development [6,7,8].

Vitamin D is an immunomodulatory hormone that has been extensively studied in relation to pregnancy complications, including preeclampsia [9,10,11,12]. Vitamin D receptors are expressed in the uterus and placenta, where vitamin D may influence maternal immune tolerance and trophoblast function [13,14]. Previous studies have focused on maternal vitamin D during early and mid-pregnancy, and many have reported associations with lower total 25(OH)D levels and increased risk of preeclampsia [8,15,16,17]. A recent study found that maternal preconception 25(OH)D deficiency, but not 25(OH)D measured at 8 weeks of gestation, was associated with preeclampsia, highlighting the preconception period as a potentially important window [10]. In addition, the study reported a U-shaped association with maternal total 25(OH)D and risk of preeclampsia, with higher risk observed at both low and elevated concentrations, compared with intermediate concentrations [10]. Calcium supplementation and vitamin D supplementation have also been evaluated as preventative strategies, specifically within populations with low dietary calcium intake [18,19]. However, findings for vitamin D supplementation and preeclampsia have been mixed, possibly because of differences in baseline vitamin D status, calcium intake, supplement dose and timing, or reliance on total 25(OH)D rather than free or bioavailable fractions [11,17,20,21].

However, emerging research also suggests that paternal preconception health may be associated with adverse pregnancy outcomes, potentially through sperm epigenetics and gene expression pathways relevant to early placental development [6,7,8,15,16]. Although maternal vitamin D has been extensively studied, limited data are available on maternal and paternal preconception total 25(OH)D and associated biomarkers in relation to preeclampsia [8,16,22,23,24].

This study investigates the association between preconception 25(OH)D serum levels and associated biomarkers in both partners and the odds of preeclampsia. By evaluating preconception biomarkers in both partners, this study aimed to characterize potentially distinct maternal and paternal patterns and to inform future research on preconception determinants of placental health.

## 2. Materials and Methods

**Figure 1:**
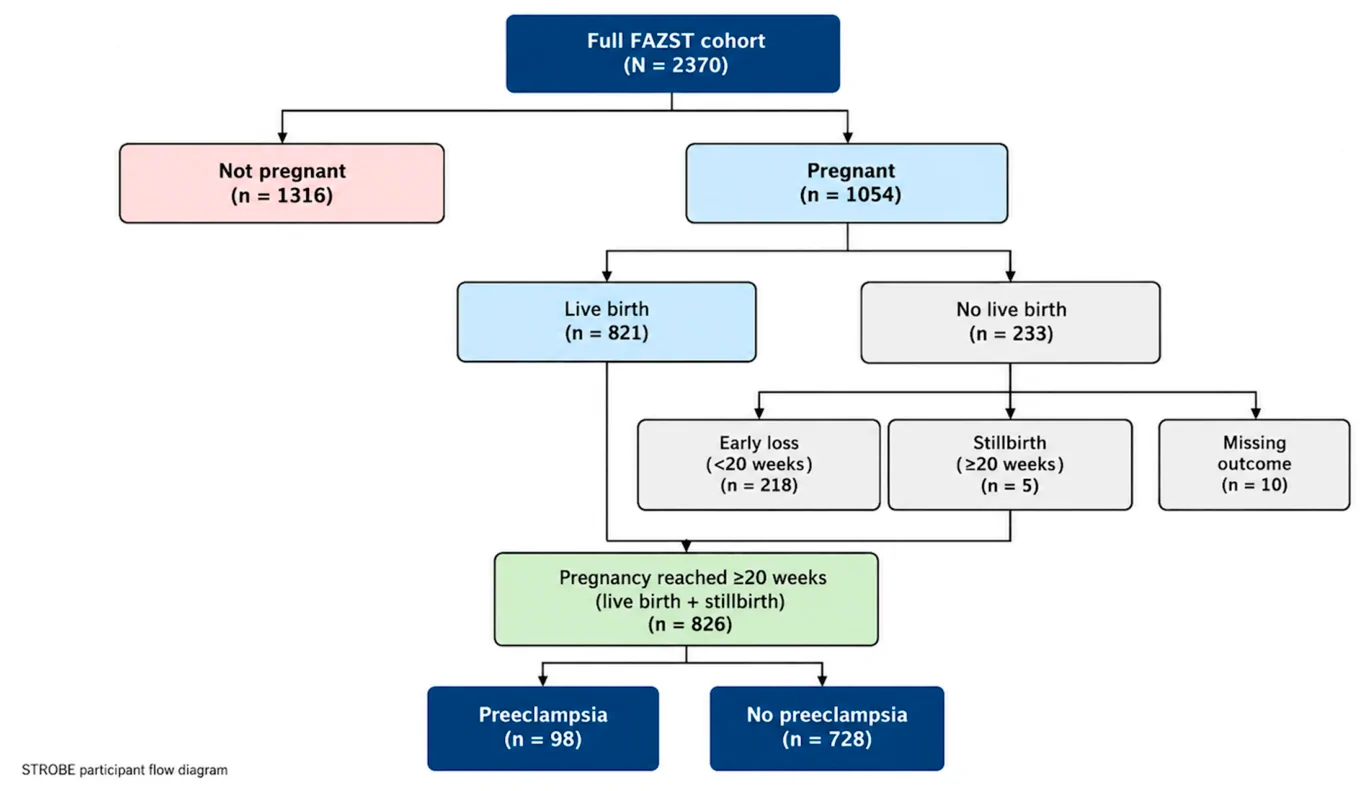
STROBE-style flow diagram showing derivation of the FAZST preeclampsia analytic population.

This secondary prospective cohort analysis used data from the Folic Acid and Zinc Supplementation Trial (FAZST), a multicenter, double-blind, block-randomized, placebo-controlled trial conducted between 2013 and 2017 to evaluate the effect of folic acid and zinc supplementation on semen quality [25]. Female partners were followed longitudinally to assess the impact of diet, lifestyle, and exercise (IDEAL) on pregnancy and live birth outcomes [26]. Eligibility included a male partner who was above 18 years of age and a female partner who was between the ages of 18 to 45 years who were in a committed relationship seeking infertility treatment [25]. Male partners in FAZST were randomized to either folic acid and zinc or placebo with a 6-month duration of participation that included a baseline visit, and follow-up visits at 2, 4 and 6 months [25]. Female partners completed a baseline visit, monthly online questionnaires, and follow-up for up to 9 months or throughout pregnancy, for a maximum participation period of 18 months [25,26]. Pregnancy was ascertained based on a positive pregnancy test and clinical confirmation from medical record abstraction. For analysis, Consolidated Standards of Reporting Trials (CONSORT) guidance was followed from the parent randomized trial, and a Strengthening the Reporting of Observational Studies in Epidemiology (STROBE)-style participant flow was used to document derivation of the secondary analysis cohort [27,28]. Of the 2370 couples enrolled in FAZST, 1054 female partners achieved a documented pregnancy during follow-up. Of these pregnancies, 821 resulted in live birth, 218 ended before 20 weeks’ gestation, 5 resulted in stillbirth at ≥20 weeks, and 10 had missing gestational outcome information. Because preeclampsia can only occur in pregnancies progressing beyond 20 weeks, the primary analytic population was restricted to 826 pregnancies that reached ≥20 weeks, comprising 821 live births and 5 stillbirths. Pregnancies ending before 20 weeks and pregnancies with missing gestational outcome information were excluded and were not classified as non-preeclampsia. Among the 826 eligible pregnancies, 98 developed preeclampsia and 728 did not. The derivation of the analytic population is shown in Figure 1.

### 2.1. Ascertainment of Vitamin D and Associated Biomarkers 

The primary biomarkers included 25(OH)D, free 25(OH)D, vitamin D-binding globulin (VDBG), albumin, and bioavailable vitamin D. Serum samples of 25(OH)D, VDBG, and albumin were collected from both female and male partners during the initial baseline visit and stored at −80° C at each study site, before being analyzed at the National Institutes of Health Biorepository in Rockville, Maryland [25].

Serum total 25(OH)D was measured using a chemiluminescent microparticle immunoassay (Abbott, Illinois, USA), which has been previously validated [28]. Vitamin D-binding globulin (VDBG) was measured using liquid chromatography–tandem mass spectrometry, with interassay laboratory CVs < 3.4% [29]. Albumin was measured using Bromcresol Purple (Roche Diagnostics, Indianapolis, IN) on a Roche COBAS 6000 chemistry analyzer (CV, 2.6%). Free and bioavailable 25(OH)D concentrations were calculated using equations developed by Powe et al. that incorporated total 25(OH)D, VDBG, and albumin concentrations [30].

### 2.2. Preeclampsia Ascertainment

Preeclampsia was ascertained from medical records based on a physician-documented diagnosis during hospital visits or at delivery. The medical record abstraction definition required new-onset hypertension at ≥20 weeks’ gestation, defined as systolic blood pressure ≥140 mm Hg or diastolic blood pressure ≥90 mm Hg, together with proteinuria. Proteinuria was defined as ≥2+ on a dipstick or a record of ≥300 mg in a 24 h urine collection [31]. Preeclampsia was analyzed as a binary outcome (yes/no). Preeclampsia status was evaluated only among pregnancies that progressed to ≥20 weeks’ gestation; pregnancies ending before 20 weeks were considered ineligible for outcome ascertainment rather than classified as non-cases.

### 2.3. Statistical Analysis

Baseline information was collected for both female and male partners. Potential confounders were identified a priori based on the prior literature and causal considerations. Variables evaluated included maternal and paternal age, body mass index (BMI), race and ethnicity, education, employment, household income, physical activity, smoking, supplement use, male randomized treatment assignment, parity, time to conception, fertility treatment type, and female and male infertility diagnoses [25,26]. Height and weight were measured at enrollment and used to calculate BMI. Blood pressure, blood, urine, and saliva samples were all collected and processed at the baseline visits [25,26].

Maternal total 25(OH)D was categorized as ≤20, >20–<30, 30–<50, and ≥50 ng/mL, with 30–<50 ng/mL serving as the reference category. Paternal total 25(OH)D was categorized as ≤20, >20–<30, and ≥30 ng/mL. Maternal free 25(OH)D was categorized as ≤8.25, >8.25–<12.5, and ≥12.5 pg/mL, while paternal free 25(OH)D was categorized as ≤8.25 versus >8.25 pg/mL. Maternal bioavailable 25(OH)D was categorized as ≤3.5, >3.5–<5.0, and ≥5.0 ng/mL, while paternal bioavailable 25(OH)D was categorized as ≤3.5 versus >3.5 ng/mL. Thresholds for VDBG, albumin, free 25(OH)D, and bioavailable 25(OH)D were evaluated using ROC curves and the Youden index to identify thresholds of discrimination for preeclampsia. We compared these data-driven thresholds with values supported by the prior literature and used them as clinically interpreted cut points when discordant, retaining the data-driven values as sensitivity checks.

Descriptive analyses characterized maternal and paternal preconception total serum 25(OH)D status and baseline characteristics. Characteristics evaluated across total 25(OH)D categories included age, race and ethnicity, parity, marital status, annual household income, physical activity, BMI, time to conception, health insurance, fertility health insurance, male treatment group, female and male infertility diagnosis, type of fertility treatment and Healthy Eating Index (HEI) (Table 1).

Multivariable logistic regression models were used to estimate odds ratios (ORs), adjusted odds ratios (aORs), and 95% CIs for associations between preconception vitamin D biomarkers and preeclampsia. All models were restricted to the 826 pregnancies that progressed to ≥20 weeks of gestation and were eligible for preeclampsia ascertainment. Biomarkers were modeled categorically using the prespecified or ROC-informed cut points described above. Primary adjusted models included maternal age, paternal age, maternal body mass index, paternal body mass index, maternal race and ethnicity, paternal race and ethnicity, maternal education, paternal education, household income, and the corresponding partners’ serum calcium concentration. Maternal biomarker models included maternal serum calcium, paternal biomarker models included paternal serum calcium, and mutually adjusted maternal–paternal models included serum calcium concentrations from both partners. Corresponding maternal and paternal biomarkers were also included together in mutually adjusted models. Multiple imputation was used for missing covariate and exposure data (ranging from 0 to 8); eligibility and preeclampsia outcome status were not imputed. No formal adjustment for multiple comparisons was applied; therefore, the reported *p*-values are nominal and were interpreted together with the magnitude and precision of the estimates. All analyses were performed using SAS version 9.4 (SAS Institute Inc., Cary, NC, USA) and R version 4.5.1.

### 2.4. Ethical Approval

The study protocol was approved by the institutional review board at each participating clinical center, and all participants provided written informed consent before enrollment [25,26]. FAZST was monitored and overseen by an independent Data Safety and Monitoring Board [25,26]. More details on the study design have been published elsewhere [25,26]. The trial was registered at clinicaltrials.gov: no. NCT01857310.

## 3. Results

### 3.1. Descriptive Analyses

Table 1 summarizes baseline characteristics of the 1054 couples according to maternal and paternal preconception total 25(OH)D status. Of the 1054 documented pregnancies, 821 resulted in live birth, 218 ended before 20 weeks of gestation, 5 resulted in stillbirth at ≥20 weeks, and 10 had missing gestational outcome information. The final analytic population therefore included 826 pregnancies that progressed to ≥20 weeks and were eligible for preeclampsia ascertainment. Among these pregnancies, 98 developed preeclampsia and 728 did not. Among females, 17% (*n* = 179) were vitamin D-deficient (≤20 ng/mL), 33% (*n* = 347) were vitamin D-insufficient (21–29 ng/mL), and 42% (*n* = 438) were vitamin D-sufficient (≥30 ng/mL). Females with deficiency had the highest mean BMI (31.3 kg/m^2^) compared to the insufficient (28.0 kg/m^2^) and sufficient (25.5 kg/m^2^) groups. Similarly, males with vitamin D deficiency (*n* = 315) had a higher mean BMI (31.6 kg/m^2^) than those who were vitamin D-insufficient (28.6 kg/m^2^) or -sufficient (27.6 kg/m^2^). Live birth was more common among vitamin D-sufficient females (78.3%, *n* = 343) than those who were vitamin D-deficient (76.5%, *n* = 137), and a similar trend was observed in males (sufficient: 75.3%, *n* = 229; deficient: 77.5%, *n* = 244). Education levels varied by vitamin D status, with 47% of females in the sufficient group holding a bachelor’s degree or higher compared to 41% in the deficient group. Household income ≥$100,000 was reported by 33% of vitamin D-sufficient females versus 22% of those who were vitamin D-deficient. High physical activity was reported by 34.0% of vitamin D-sufficient females compared with 21.8% of vitamin D-deficient females, while among males, 35.2% of vitamin D-sufficient participants reported high physical activity compared with 27.3% of vitamin D-deficient participants. Additionally, diet quality, measured using the Healthy Eating Index (HEI), increased with vitamin D status in both females (60.5 deficient vs. 62.5 sufficient) and males (56.9 deficient vs. 60.0 sufficient). Vitamin D-sufficient participants were also more likely to have received IVF treatment compared with vitamin D-deficient participants (females: 34.5% vs. 24.0%; males: 31.3% vs. 25.1%). Female infertility diagnoses were more common among vitamin D-deficient participants (41.9%) than among those who were vitamin D-sufficient (24.9%). Vitamin D-deficient participants also reported slightly longer durations of attempting conception than vitamin D-sufficient participants (Table 1).

Biomarker measurements were available for approximately 91% of female partners and 94% of male partners (Table 2). Mean total 25(OH)D was higher among females (30.16 ± 12.00) versus males (25.86 ± 10.28 ng/mL). In addition, males were more vitamin D-deficient (≤20 ng/mL at 29.9%) and -insufficient (21–<29 ng/mL at 35.8%), while women were less vitamin D-deficient (≤20 ng/mL at 17.0%) and -insufficient (21–<29 ng/mL at 32.9%). However, women had higher sufficient (30–<50 ng/mL at 35.9%) and elevated levels (≥50 ng/mL at 5.7%) of serum 25(OH)D compared to males with sufficient (30–<50 ng/mL at 26.3%) and elevated levels (≥50 ng/mL at 2.6%). Females had higher VDBG levels (254 µg/mL vs. 238 µg/mL), while more males had lower levels <240 µg/mL (53% vs. 38%). Albumin was higher in males (4.67 g/dL vs. 4.51 g/dL), and low albumin (<4.5 g/dL) was less common in males (17%) vs. females (38%). For vitamin D-associated biomarkers, men had lower free and bioavailable 25(OH)D (free: 8.0 pg/mL vs. 8.9 pg/mL; bioavailable: 3.37 ng/mL vs. 3.63 ng/mL), with >55% of males in the low categories vs. ~45–50% of females. Overall, males showed lower total, free, and bioavailable 25(OH)D and lower VDBG, whereas females more often met sufficiency thresholds but had lower albumin.

To further characterize biomarker distributions across study characteristics, Supplementary Table S1 includes maternal preconception vitamin D biomarker distributions by preeclampsia status, male folic acid/zinc or placebo treatment assignment, and the maximum fertility treatment type used by the couple. Supplementary Table S2 includes the paternal preconception vitamin D biomarker distributions by preeclampsia status, male folic acid/zinc or placebo treatment assignment, and the maximum fertility treatment type used by the couple.

### 3.2. Logistic Regression Analyses

Among female partners, categorical estimates for total 25(OH)D were consistent with a possible U-shaped pattern with preeclampsia (Table 3). Compared with concentrations of 30–<50 ng/mL, both deficient (≤20 ng/mL; aOR = 2.58, 95% CI: 1.13–5.88) and elevated concentrations (≥50 ng/mL; aOR = 5.45, 95% CI: 1.71–17.41) were associated with increased odds of preeclampsia after adjustment for potential confounders. Concentrations >20–<30 ng/mL were not statistically significantly associated with preeclampsia (aOR = 1.84, 95% CI: 0.92–3.68). Similar categorical patterns were observed for maternal free and bioavailable 25(OH)D. Compared with free 25(OH)D concentrations >8.25–<12.5 pg/mL, concentrations ≤8.25 pg/mL (aOR = 2.23, 95% CI: 1.13–4.43) and ≥12.5 pg/mL (aOR = 4.71, 95% CI: 1.97–11.29) were associated with higher odds of preeclampsia. Compared with bioavailable 25(OH)D concentrations >3.5–<5.0 ng/mL, concentrations ≤3.5 ng/mL (aOR = 2.61, 95% CI: 1.19–5.72) and ≥5.0 ng/mL (aOR = 5.38, 95% CI: 2.15–13.46) were associated with higher odds of preeclampsia.

Among male partners, total 25(OH)D was not associated with preeclampsia. Lower paternal bioavailable 25(OH)D concentrations (≤3.5 ng/mL) were associated with reduced odds of preeclampsia compared with concentrations >3.5 ng/mL (aOR = 0.50, 95% CI: 0.28–0.89). Although lower paternal free 25(OH)D concentrations demonstrated a similar inverse direction, the association did not reach statistical significance (aOR = 0.55, 95% CI: 0.30–1.02). Maternal and paternal VDBG and albumin were not significantly associated with preeclampsia in adjusted analyses.

Results from mutually adjusted models were similar to the primary analyses (Supplementary Table S3), indicating that the observed associations between maternal total, free, and bioavailable 25(OH)D concentrations and preeclampsia were largely independent. Additionally, further sensitivity analyses were conducted to assess the robustness of our results by including inverse probability-weighted analyses, yielding findings that were attenuated but consistent with our primary results (Supplementary Table S4).

## 4. Discussion

In this secondary cohort analysis restricted to pregnancies progressing to ≥20 weeks, maternal preconception total, free, and bioavailable 25(OH)D showed patterns consistent with a possible U-shaped relationship with preeclampsia [10]. Compared with intermediate concentrations, both deficient levels ≤20 ng/mL and elevated levels ≥50 ng/mL of total 25(OH)D were associated with higher odds of preeclampsia. Importantly, free and bioavailable 25(OH)D concentrations showed larger associations with both lower and higher concentrations than the corresponding estimates for total 25(OH)D. These results further suggest that biomarkers integrating binding proteins may serve as more physiologically relevant predictors of preeclampsia than total 25(OH)D concentrations alone. The points we identified were ≤8.25 pg/mL and ≥12.5 pg/mL for free 25(OH)D, and ≤3.5 ng/mL and ≥5 ng/mL for bioavailable 25(OH)D concentrations, which also highlight that both low and elevated levels confer a risk of preeclampsia. In contrast to the U-shaped associations observed in female partners, lower levels of bioavailable 25(OH)D concentrations in male partners were consistently associated with a reduced risk of preeclampsia.

Potential biological mechanisms suggest that maternal and paternal preconception health, specifically for micronutrient status like vitamin D and associated biomarkers, may contribute to the trajectory of the health of the pregnancy before conception. These findings motivate further dual-partner research examining whether paternal preconception factors may contribute to placental development [8]. Deficient 25(OH)D levels may impair trophoblast invasion and angiogenesis, while elevated levels may contribute to dysregulated immune responses or altered placental gene expression, disrupting implantation and placentation [32,33]. These dual risks support the hypothesis that maintaining an optimal range of vitamin D and associated biomarker levels prior to conception may be biologically supportive in reducing the odds of preeclampsia, a finding which is consistent with prior research that examined reproductive and perinatal outcomes [9]. Additionally, the previous literature has shown that a higher threshold of albumin appears to be vital for maintaining optimal vascular function and endothelial health, which are critical in the pathophysiology of preeclampsia [34,35]. Albumin also plays an important role in preventing excess fluid retention, which helps regulate both vascular tone and blood pressure [36,37]. The previous literature has concurrently indicated that inadequate albumin levels are associated with an increased risk of preeclampsia [37,38]. Additionally, free 25(OH)D and bioavailable 25(OH)D integrate circulating total 25(OH)D with both albumin and VDBG concentrations, and represent more hormonally active 25(OH)D levels in the body that are accessible to tissues when biologically needed [36]. However, neither VDBG nor albumin was independently associated with preeclampsia in the adjusted models. Accordingly, the associations observed for calculated free and bioavailable 25(OH)D suggest that these measures may warrant further evaluation as potential biomarkers; however, the present study did not assess their clinical predictive performance.

Evidence for paternal vitamin D biomarkers was limited. Paternal total 25(OH)D and free 25(OH)D were not statistically significantly associated with preeclampsia. Lower paternal bioavailable 25(OH)D was associated with lower odds of preeclampsia in the fully adjusted model; however, this isolated paternal finding should be considered exploratory and requires external validation. Because all couples in FAZST were seeking infertility treatment, our findings should be interpreted within the context of fertility treatment-related preeclampsia risk. IVF treatment was used for 28.1% of the participants in the trial and was more common among vitamin D-sufficient than vitamin D-deficient females. Prior studies have reported higher risks of hypertensive disorders of pregnancy and preeclampsia diagnoses after IVF or ICSI use compared to spontaneous conception [39,40]. Therefore, these associations may reflect underlying infertility diagnoses that may not have been accounted for, despite adjusting for maternal, paternal, and sociodemographic characteristics.

The 2024 Endocrine Society guideline suggests empiric vitamin D supplementation during pregnancy and advises against routine 25(OH)D testing among generally healthy pregnant individuals. The trials informing this recommendation used vitamin D doses ranging from 600 to 5000 IU/day equivalents, with a weighted average of approximately 2500 IU/day [41,42]. These recommendations do not account for women who may have elevated levels of total 25(OH)D and those with a history of preeclampsia. The inclusion of paternal biomarkers identifies an important area for future preconception research but does not support routine partner screening or treatment decisions. Therefore, the present observational findings should not be used to recommend routine partner screening, to define treatment thresholds, or to alter current supplementation practices. Instead, they identify questions for future preconception research.

Future studies should evaluate maternal and paternal total, free, and bioavailable 25(OH)D in more diverse populations. Longitudinal studies with repeated biomarker measurements are needed to clarify temporal patterns and potential immunologic, epigenetic, and placental pathways. Intervention studies would be required to determine whether modifying preconception vitamin D status changes preeclampsia risk, particularly among individuals with a history of preeclampsia or other established risk factors.

### 4.1. Strengths and Limitations

This study has several strengths, including prospective preconception enrollment, paired biomarker measurements from both partners, and assessment of total, free, and bioavailable 25(OH)D. This study has several limitations. First, this was a secondary observational analysis; therefore, causal inferences cannot be made, and residual confounding remains possible despite adjustment for maternal, paternal, and sociodemographic characteristics. Second, the cohort consisted of couples seeking infertility treatment, which limits generalizability to couples conceiving without treatment. Third, preeclampsia was analyzed as a binary outcome and could not have been further classified into early-onset versus late-onset preeclampsia or severe versus non-severe disease. These subtypes may have different underlying pathophysiology pathways. Fourth, vitamin D status may reflect broader health-related behaviors and exposures, including outdoor activity, diet, and sun exposure, as well as the use of vitamin D-containing nutritional supplements such as prenatal vitamins, that could not be fully captured. Fifth, biomarkers were measured at a single preconception point, and within-person changes could not be evaluated. Sixth, several biomarker cut points were data-informed and required external validation. Seventh, we evaluated multiple correlated vitamin D biomarkers in both partners using several categorical comparisons and model specifications without formal adjustment for multiple comparisons. Consequently, the possibility of type I error cannot be excluded, particularly for isolated or borderline paternal findings. The paternal results should therefore be considered exploratory and require external validation. Finally, most participants were Non-Hispanic White and had relatively high educational attainment, which limits generalizability to more racially, ethnically, socioeconomically, and geographically diverse populations.

### 4.2. Conclusions

This study provides evidence that, among pregnancies progressing to ≥20 weeks in couples seeking infertility treatment, lower and higher categories of maternal total, free, and bioavailable 25(OH)D were associated with higher odds of preeclampsia relative to intermediate categories. These categorical findings were consistent with a possible U-shaped pattern, although nonlinearity was not formally tested. Lower paternal bioavailable 25(OH)D was associated with lower odds of preeclampsia in the fully adjusted model; however, this isolated finding should be considered exploratory because of multiple comparisons and requires external validation. Given the observational design, potential residual confounding, and limited generalizability of this infertility-treatment cohort, these findings should not be used to define screening or supplementation thresholds.

## Figures and Tables

**Table 1:** Baseline characteristics of female and male partners according to preconception serum 25(OH)D status in FAZST (N = 1054).

	Female				Male			
Total	Overall (%)	25(OH)D-Deficient (≤20 ng/mL)	25(OH)D-Insufficient (>20–<30 ng/mL)	25(OH)D-Sufficient (≥30 ng/mL)	Overall (%)	25(OH)D-Deficient (≤20 ng/mL)	25(OH)D-Insufficient (>20–<30 ng/mL)	25(OH)D-Sufficient (≥30 ng/mL)
	N = 1054	N = 179	N = 347	N = 438	N = 1054	N = 315	N = 377	N = 304
Age, years	30.3 (4.7)	30.0 (4.9)	29.9 (4.7)	30.7 (4.5)	32.0 (5.5)	32.0 (5.4)	31.8 (5.3)	32.4 (5.6)
BMI, kg/m^2^	27.6 (7.6)	31.3 (10.0)	28.0 (6.9)	25.5 (6.1)	29.4 (6.3)	31.6 (7.1)	28.6 (5.6)	27.6 (5.1)
BMI Category (kg/m^2^)								
Underweight (<18.5)	37 (3.5)	4 (2.2)	11 (3.2)	17 (3.9)	5 (0.5)	1 (0.3)	2 (0.5)	0 (0.0)
Healthy weight (18.5–24.9)	464 (44.0)	57 (31.8)	139 (40.1)	243 (55.5)	266 (25.2)	47 (14.9)	111 (29.4)	100 (32.9)
Overweight (25.0–29.9)	244 (23.2)	35 (19.6)	86 (24.8)	95 (21.7)	371 (35.2)	104 (33.0)	126 (33.4)	127 (41.8)
Obese (>30.0)	309 (29.3)	83 (46.4)	111 (32.0)	83 (19.0)	412 (39.1)	163 (51.8)	138 (36.6)	77 (25.3)
Race and Ethnicity								
Non-Hispanic White	893 (84.7)	122 (68.2)	297 (85.6)	403 (92.0)	871 (82.6)	226 (71.8)	328 (87.0)	273 (89.8)
Black or African American	13 (1.2)	9 (5.0)	3 (0.9)	1 (0.2)	17 (1.6)	7 (2.2)	7 (1.9)	1 (0.3)
Asian	63 (6.0)	24 (13.4)	18 (5.2)	14 (3.2)	46 (4.4)	22 (7.0)	13 (3.5)	8 (2.6)
Hispanic/Latinx	44 (4.2)	9 (5.0)	18 (5.2)	11 (2.5)	61 (5.8)	31 (9.8)	16 (4.2)	11 (3.6)
Other Race/Ethnicity	33 (3.1)	14 (7.8)	9 (2.6)	6 (1.4)	53 (5.0)	26 (8.3)	12 (3.2)	10 (3.3)
Education								
≤High School	96 (9.1)	25 (14.0)	34 (9.8)	29 (6.6)	129 (12.2)	40 (12.7)	46 (12.2)	33 (10.9)
Some College	304 (28.8)	58 (32.4)	108 (31.1)	108 (24.7)	332 (31.5)	108 (34.3)	116 (30.8)	87 (28.6)
Bachelor’s Degree	401 (38.1)	48 (26.8)	133 (38.3)	187 (42.7)	348 (33.0)	88 (27.9)	142 (37.7)	105 (34.5)
≥Master’s Degree	241 (22.9)	47 (26.3)	72 (20.8)	109 (24.9)	236 (22.4)	78 (24.8)	71 (18.8)	75 (24.7)
Employment								
Full-Time	640 (60.7)	106 (59.2)	207 (59.7)	276 (63.0)	739 (70.1)	230 (73.0)	249 (66.1)	227 (74.7)
Part-Time	165 (15.7)	28 (15.6)	53 (15.3)	67 (15.3)	52 (4.9)	19 (6.0)	17 (4.5)	11 (3.6)
Student	26 (2.5)	7 (3.9)	7 (2.0)	11 (2.5)	80 (7.6)	19 (6.0)	38 (10.1)	15 (4.9)
Not Employed	163 (15.5)	26 (14.5)	56 (16.1)	65 (14.8)	124 (11.8)	30 (9.5)	52 (13.8)	35 (11.5)
Household Income								
<$40,000	118 (11.2)	23 (12.9)	45 (13.0)	40 (9.1)	120 (11.4)	41 (13.0)	45 (11.9)	16 (5.3)
$40,000–<$75,000	363 (34.4)	67 (37.4)	133 (38.3)	130 (29.7)	358 (34.0)	115 (36.5)	122 (32.4)	27 (8.9)
$75,000–<$100,000	242 (23.0)	39 (21.8)	77 (22.2)	109 (24.9)	243 (23.1)	74 (23.5)	94 (24.9)	100 (32.9)
≥$100,000	275 (26.1)	40 (22.4)	78 (22.5)	145 (33.1)	277 (26.3)	74 (23.5)	102 (27.1)	68 (22.4)
Married								
Yes	1013 (96.1)	175 (97.8)	329 (94.8)	420 (95.9)	1013 (96.1)	305 (96.8)	362 (96.0)	289 (95.1)
No	40 (3.8)	4 (2.2)	18 (5.2)	18 (5.2)	39 (3.7)	10 (3.2)	14 (3.7)	15 (4.9)
Fertility Insurance								
Yes	337 (32.0)	62 (34.6)	108 (31.1)	145 (33.1)	281 (26.7)	91 (28.9)	96 (25.5)	84 (27.6)
No	448 (42.5)	64 (35.8)	148 (42.7)	193 (44.1)	446 (42.3)	130 (41.3)	173 (45.9)	125 (41.1)
Don’t Know	239 (22.7)	44 (24.6)	83 (23.9)	91 (20.8)	282 (26.8)	83 (26.4)	95 (25.2)	82 (27.0)
Smoking								
Yes	41 (3.9)	7 (3.9)	15 (4.3)	16 (3.7)	76 (7.2)	30 (9.5)	18 (4.8)	25 (8.2)
No	957 (90.8)	161 (89.9)	308 (88.8)	406 (92.7)	923 (87.6)	270 (85.7)	341 (90.5)	262 (86.2)
Live Birth								
Yes	821 (77.9)	137 (76.5)	272 (78.4)	343 (78.3)	821 (77.9)	244 (77.5)	307 (81.4)	229 (75.3)
No	233 (22.1)	42 (23.5)	75 (21.6)	95 (21.7)	233 (22.1)	71 (22.5)	70 (18.6)	75 (24.7)
Early Loss (<20 wks)	218 (20.7)	38 (21.2)	72 (20.7)	90 (20.5)	218 (20.7)	65 (20.6)	66 (17.5)	71 (23.3)
Stillbirth (≥20 wks)	5 (0.5)	0 (0.0)	0 (0.0)	3 (0.7)	5 (0.5)	1 (0.3)	2 (0.5)	2 (0.7)
Missing	10 (0.9)				10 (0.9)			
Physical Activity								
Low	330 (31.3)	73 (40.8)	98 (28.2)	123 (28.1)	321 (30.5)	121 (38.4)	113 (30.0)	70 (23.0)
Moderate	347 (32.9)	55 (30.7)	119 (34.3)	147 (33.6)	353 (33.5)	91 (28.9)	139 (36.9)	111 (36.5)
High	317 (30.1)	39 (21.8)	106 (30.6)	149 (34.0)	321 (30.5)	86 (27.3)	104 (27.6)	107 (35.2)
Fertility Treatment Received								
IUI	227 (21.5)	45 (25.1)	71 (20.5)	96 (21.9)	227 (21.5)	70 (22.2)	82 (21.8)	67 (22.0)
IVF	296 (28.1)	43 (24.0)	88 (25.4)	151 (34.5)	296 (28.1)	79 (25.1)	113 (30.0)	95 (31.3)
None	265 (25.1)	42 (23.5)	95 (27.4)	97 (22.2)	265 (25.1)	76 (24.1)	92 (24.4)	77 (25.3)
OI Only	266 (25.2)	49 (27.4)	93 (26.8)	94 (21.5)	266 (25.2)	90 (28.6)	90 (23.9)	65 (21.4)
Infertility Dx								
Yes	250 (23.7)	75 (41.9)	83 (23.9)	109 (24.9)	135 (12.8)	34 (10.8)	51 (13.5)	44 (14.5)
No	413 (39.2)	40 (22.4)	133 (38.3)	169 (38.6)	529 (50.2)	175 (55.6)	199 (52.8)	133 (43.8)
Treatment Assignment								
Folic Acid/Zinc	519 (49.2)	82 (45.8)	164 (47.3)	219 (50.0)	519 (49.2)	152 (48.3)	184 (48.8)	149 (49.0)
Placebo	535 (50.8)	97 (54.2)	183 (52.7)	219 (50.0)	535 (50.8)	163 (51.8)	193 (51.2)	155 (51.0)
Parity								
0	648 (61.5)	120 (67.0)	218 (62.8)	259 (59.1)	648 (61.5)	204 (64.8)	236 (62.6)	174 (57.2)
≥1	351 (33.3)	52 (29.1)	113 (32.6)	153 (34.9)	351 (33.3)	95 (30.2)	125 (33.2)	108 (35.5)
Time Trying to Conceive, months	25.2 (23.5)	27.3 (26.2)	25.4 (23.3)	24.5 (22.8)	25.2 (23.5)	25.8 (23.9)	24.8 (23.5)	24.6 (22.5)
HEI Total Score	61.4 (9.4)	60.5 (9.4)	60.7 (9.4)	62.5 (9.6)	58.5 (9.5)	56.9 (9.9)	58.7 (9.3)	60.0 (9.0)

Note: Values are presented as *n* (%) or mean (standard deviation). Percentages were calculated using available data for each characteristic and may not sum precisely to 100% because of variable-specific missingness and rounding. Preeclampsia analyses were restricted to 826 pregnancies that progressed to ≥20 weeks of gestation.

**Table 2:** Distribution of preconception 25(OH)D status and associated biomarkers in female and male partners within FAZST (N = 1054).

Biomarker	Female Mean ± SD	Female N (%)	Male Mean ± SD	Male N (%)
Total 25(OH)D	30.16 ± 12.00	964 (91.46)	25.86 ± 10.28	996 (94.50)
Deficient (≤20 ng/mL)		179 (16.98)		315 (29.89)
Insufficient (>20–<30 ng/mL)		347 (32.92)		377 (35.77)
Sufficient (30–<50 ng/mL)		378 (35.86)		277 (26.28)
Elevated (≥50 ng/mL)		60 (5.69)		27 (2.56)
Missing		90 (8.54)		58 (5.50)
Vitamin D-Binding Globulin	254.03 ± 42.81	964 (91.46)	237.86 ± 26.53	999 (94.78)
≤240 ug/mL		405 (38.43)		561 (53.23)
240–<280 ug/mL		401 (38.05)		
≥280 ug/mL		158 (14.99)		
Missing		90 (8.54)		55 (5.22)
Albumin	4.51 ± 0.26	964 (91.46)	4.67 ± 0.25	996 (94.50)
<4.5 g/dL		395 (37.48)		181 (17.17)
≥4.5 g/dL		569(53.98)		815 (77.32)
Missing		90 (8.54)		58 (5.50)
Free 25(OH)D	8.86 ± 3.50	963 (91.34)	7.97 ± 3.13	994 (94.31)
≤8.25 pg/mL		478 (45.35)		583 (55.31)
>8.25–<12.5 pg/mL		375 (35.58)		
≥12.5 pg/mL		110 (10.44)		
Missing		91 (8.63)		60 (5.69)
Bioavailable 25(OH)D	3.63 ± 1.48	963 (91.34)	3.37 ± 1.34	994 (94.31)
≤3.5 ng/mL		524 (49.72)		584 (55.41)
>3.5–<5.0 ng/mL		314 (29.79)		
≥5.0 ng/mL		125 (11.86)		
Missing		91 (8.63)		60 (5.69)

**Table 3:** Associations between maternal and paternal preconception vitamin D biomarkers and preeclampsia among pregnancies progressing to ≥20 weeks in FAZST (N = 826).

Biomarker	Category	Unadjusted OR (95% CI)	*p*-Value	Adjusted OR (95% CI)	*p*-Value
Female Total 25(OH)D	≤20 ng/mL vs. 30–<50 ng/mL	2.63 (1.32–5.27)	0.006	2.58 (1.13–5.88)	0.024
	>20–<30 ng/mL vs. 30–<50 ng/mL	1.80 (1.00–3.24)	0.049	1.84 (0.92–3.68)	0.086
	≥50 ng/mL vs. 30–<50 ng/mL	4.07 (1.55–10.66)	0.005	5.45 (1.71–17.41)	0.004
Male Total 25(OH)D	≤20 ng/mL vs. ≥30 ng/mL	0.86 (0.46–1.60)	0.637	0.61 (0.28–1.33)	0.213
	>20–<30 ng/mL vs. ≥30 ng/mL	0.94 (0.54–1.65)	0.839	0.72 (0.37–1.39)	0.330
Female Free 25(OH)D	≤8.25 pg/mL vs. >8.25–<12.5 pg/mL	2.48 (1.41–4.36)	0.002	2.23 (1.13–4.43)	0.021
	≥12.5 pg/mL vs. >8.25–<12.5 pg/mL	3.79 (1.81–7.93)	<0.001	4.71 (1.97–11.29)	<0.001
Male Free 25(OH)D	≤8.25 pg/mL vs. >8.25 pg/mL	0.80 (0.49–1.30)	0.368	0.55 (0.30–1.02)	0.056
Female Bioavailable 25(OH)D	≤3.5 ng/mL vs. >3.5–<5.0 ng/mL	3.30 (1.67–6.55)	<0.001	2.61 (1.19–5.72)	0.017
	≥5.0 ng/mL vs. >3.5–<5.0 ng/mL	4.89 (2.25–10.64)	<0.001	5.38 (2.15–13.46)	<0.001
Male Bioavailable 25(OH)D	≤3.5 ng/mL vs. >3.5 ng/mL	0.68 (0.43–1.08)	0.104	0.50 (0.28–0.89)	0.019
Female VDBG	≤240 µg/mL vs. >240–<280 µg/mL	1.10 (0.67–1.83)	0.699	1.39 (0.77–2.53)	0.274
	≥280 µg/mL vs. >240–<280 µg/mL	1.45 (0.75–2.81)	0.267	1.10 (0.47–2.56)	0.821
Male VDBG	<240 µg/mL vs. ≥240 µg/mL	0.70 (0.44–1.12)	0.140	0.61 (0.34–1.10)	0.100
Female Albumin	<4.5 g/dL vs. ≥4.5 g/dL	1.73 (1.02–2.94)	0.044	1.36 (0.72–2.58)	0.342
Male Albumin	<4.5 g/dL vs. ≥4.5 g/dL	0.88 (0.44–1.73)	0.705	0.76 (0.35–1.64)	0.480

All models were restricted to pregnancies progressing to ≥20 weeks of gestation (N = 826, 98 preeclampsia cases and 728 non-cases). Adjusted models include maternal age, paternal age, maternal body mass index, paternal body mass index, maternal race and ethnicity, paternal race and ethnicity, maternal education, paternal education, household income, and the corresponding partners’ serum calcium concentration. Corresponding maternal and paternal biomarkers were included simultaneously in each model. Missing exposure and covariate data were addressed using multiple imputation with 20 imputed datasets.

## Data Availability

The data underlying this article have been provided by the Eunice Kennedy Shriver National Institute of Child Health and Human Development (NICHD), National Institutes of Health, under a data use agreement. We do not have permission to share it with other individuals, within or outside the primary author’s institution, or with commercial enterprises. Data from the FAZST study are available to approved researchers through the data use agreement. Information about the study and data are available here: https://www.nichd.nih.gov/about/org/dir/dph/officebranch/eb/folic-acid-zinc (accessed on 13 June 2026). Data used in this study were created using pre-existing questionnaires, and variables derived from this data were previously returned to NICHD contacts for this project.

## Supplementary Materials

The following supporting information can be downloaded at: https://www.mdpi.com/article/10.3390/nu18162597/s1. Table S1: Distribution of Maternal Preconception Vitamin D Biomarkers by Preeclampsia Status, Male Treatment Assignment, and Maximum Fertility Treatment Type Received in the FAZST Trial; Table S2: Distribution of Paternal Preconception Vitamin D Biomarkers by Preeclampsia Status, Male Treatment Assignment, and Maximum Fertility Treatment Type Received in the FAZST Trial; Table S3: Mutually Adjusted Associations of Maternal and Paternal Total 25(OH)D, Vitamin D Binding Protein, and Albumin with Odds of Preeclampsia in the FAZST Trial; Table S4: Fully Adjusted Single-Model Associations Between Maternal and Paternal Preconception Vitamin D Biomarkers and Odds of Preeclampsia with Inverse Probability Weighting (IPW) Restricted to ≥20 Weeks Gestation in the FAZST Trial.

## Abbreviations

The following abbreviations are used in this manuscript:

OR	Odds Ratio
CI	Confidence Interval
ROC	Receiver Operating Characteristic
BMI	Body Mass Index
VDBG	Vitamin D-Binding Globulin
25(OH)D	25-Hydroxyvitamin D
HEI	Healthy Eating Index
ART	Assisted Reproductive Technology
CONSORT	Consolidated Standards of Reporting Trials
STROBE	Strengthening the Reporting of Observational Studies in Epidemiology
