# Supplementary material for: Preconception Partner-Specific Vitamin D Biomarkers and Risk of Preeclampsia Among Couples Undergoing Fertility Treatment: A Secondary Cohort Analysis of FAZST
Source: Nutrients. 2026 Aug 8;18(16):2597. doi: 10.3390/nu18162597 (PMC13516339; doi:10.3390/nu18162597)
Supplement: Supplementary file 1 [file nutrients-18-02597-s001.zip › nutrients-4406236-supplementary.pdf]

Supplementary Table S1. Distribution of Maternal Preconception Vitamin D Biomarkers by Preeclampsia Status, Male Treatment Assignment, and Maximum Fertility Treatment Type Received in the FAZST Trial

	**Preeclampsia**		**Male Treatment Assignment**		**Max Fertility Treatment**			
	Yes<br>N (%)	No<br>N (%)	Folic Acid/Zinc<br>N (%)	Placebo<br>N (%)	IVF<br>N (%)	IUI<br>N (%)	OI only<br>N (%)	None<br>N (%)
**Total 25(OH)D**	90	874	465	499	282	212	236	234
**Deficient (<20 ng/mL)**	23 (25.56)	156 (17.85)	82 (17.63)	97 (19.44)	43 (15.25)	45 (21.23)	49 (20.76)	42 (17.95)
**Insufficient (20-<29 ng/mL)**	34 (37.78)	313 (35.81)	164 (35.27)	183 (36.67)	88 (31.21)	71 (33.49)	93 (39.41)	95 (40.60)
**Sufficient (30-<50 ng/mL)**	23 (25.56)	306 (35.01)	160 (34.41)	169 (33.87)	118 (41.84)	72 (33.96)	65 (27.54)	74 (31.62)
**Elevated (>50 ng/mL)**	10 (11.11)	50 (5.72)	32 (6.88)	28 (5.61)	22 (7.80)	11 (5.19)	14 (5.93)	13 (5.56)
**Vitamin D Binding Globulin**	90	874	465	499	282	211	236	235
**<240 ug/mL**	40 (44.44)	365 (41.76)	180 (38.71)	225 (45.09)	95 (33.69)	90 (42.65)	115 (48.73)	105 (44.68)
**240-280 ug/mL**	33 (36.67)	368 (42.11)	201 (43.23)	200 (40.08)	91 (32.27)	104 (49.29)	96 (40.68)	110 (46.81)
**>280 ug/mL**	17 (18.89)	141 (16.13)	84 (18.06)	74 (14.83)	96 (34.04)	17 (8.06)	25 (10.59)	20 (8.51)
**Albumin**	90	874	465	499	282	212	236	234

**<4.5 g/dL**	46 (51.11)	349 (39.93)	184 (39.57)	211 (42.28)	126 (44.68)	92 (43.40)	94 (39.83)	83 (35.47)
**≥4.5 g/dL**	44 (48.89)	525 (60.07)	281 (60.43)	288 (57.72)	156 (55.32)	120 (56.60)	142 (60.17)	151 (64.53)
**Free 25(OH)D**	90	873	465	499	282	211	236	234
**<8.25 pg/mL**	54 (60.00)	424 (48.57)	231 (49.68)	247 (49.50)	137 (48.58)	104 (49.29)	124 (52.54)	113 (48.29)
**8.25-12.5 pg/mL**	19 (21.11)	356 (40.78)	181 (38.92)	194 (38.88)	105 (37.23)	79 (37.44)	90 (38.14)	101 (43.16)
**>12.5 pg/mL**	17 (18.89)	93 (10.65)	53 (11.40)	57 (11.42)	40 (14.18)	28 (13.27)	22 (9.32)	20 (8.55)
**Bioavailable 25(OH)D**	90	873	465	499	282	211	236	234
**<3.5 ng/mL**	59 (65.56)	465 (53.26)	253 (54.41)	271 (54.31)	154 (54.61)	116 (54.98)	133 (56.36)	121 (51.71)
**3.5-5 ng/mL**	12 (13.33)	302 (34.59)	148 (31.83)	166 (33.27)	85 (30.14)	63 (29.86)	78 (33.05)	88 (37.61)
**>5 ng/mL**	19 (21.11)	106 (12.14)	64 (13.76)	61 (12.22)	43 (15.25)	32 (15.17)	25 (10.59)	25 (10.68)

Supplementary Table S2. Distribution of Paternal Preconception Vitamin D Biomarkers by Preeclampsia Status, Male Treatment Assignment, and Maximum Fertility Treatment Type Received in the FAZST Trial

	**Preeclampsia**		**Male Treatment Assignment**		**Max Fertility Treatment**			
	Yes<br>N (%)	No<br>N (%)	Folic Acid/Zinc<br>N (%)	Placebo<br>N (%)	IVF<br>N (%)	IUI<br>N (%)	OI only<br>N (%)	None<br>N (%)
**Total 25(OH)D**	90	906	485	511	287	219	245	245
**Deficient (<20 ng/mL)**	29 (32.22)	286 (31.57)	152 (31.34)	163 (31.90)	79 (27.53)	70 (31.96)	90 (36.73)	76 (31.02)
**Insufficient (20-<29 ng/mL)**	34 (37.78)	343 (37.86)	184 (37.94)	193 (37.77)	113 (39.37)	82 (37.44)	90 (36.73)	92 (37.55)
**Sufficient (30-<50 ng/mL)**	24 (26.67)	218 (24.06)	120 (24.74)	122 (23.87)	74 (25.78)	51 (23.29)	52 (21.22)	65 (26.53)
**Elevated (>50 ng/mL)**	1 (1.11)	26 (2.87)	12 (2.47)	15 (2.94)	13 (4.53)	6 (2.74)	4 (1.63)	4 (1.63)
**Vitamin D Binding Globulin**	90	909	486	513	286	219	246	248
**<240 ug/mL**	44 (48.89)	517 (57.06)	276 (56.91)	285 (55.77)	161 (56.10)	128 (58.45)	137 (55.92)	135 (55.10)
**Albumin**	90	906	485	511	287	219	245	245
**<4.5 g/dL**	14 (15.56)	167 (18.43)	86 (17.73)	95 (18.59)	46 (16.03)	45 (20.55)	50 (20.41)	40 (16.33)

**Free 25(OH)D**	90	904	483	511	286	219	244	245
**<8.25 pg/mL**	51 (56.67)	532 (58.72)	288 (59.38)	295 (57.73)	158 (55.05)	125 (57.08)	153 (62.45)	147 (60.00)
**Bioavailable 25(OH)D**	90	904	483	511	286	219	244	245
**<3.5 ng/mL**	49 (54.44)	535 (59.05)	286 (58.97)	298 (58.32)	161 (56.10)	127 (57.99)	154 (62.86)	142 (57.96)

Supplementary Table S3. Mutually Adjusted Associations of Maternal and Paternal Total 25(OH)D, Vitamin D Binding Protein, and Albumin with Odds of Preeclampsia in the FAZST Trial

Biomarker	Comparison	Adjusted OR (95% CI)	P-value
**Female Total 25(OH)D**	≤20 vs. 30–49 ng/mL	2.24 (1.13–4.43)	0.021
	21–29 vs. 30–49 ng/mL	1.67 (0.93–3.01)	0.086
	≥50 vs. 30–49 ng/mL	2.89 (1.16–7.17)	0.022
**Female VDBG**	<240 vs. 240–280 µg/mL	1.22 (0.73–2.06)	0.443
	>280 vs. 240–280 µg/mL	1.20 (0.60–2.44)	0.604
**Female Albumin**	<4.5 vs. ≥4.5 g/dL	1.47 (0.84–2.55)	0.175
**Male Total 25(OH)D**	≤20 vs. ≥30 ng/mL	1.10 (0.59–2.04)	0.771
	21–29 vs. ≥30 ng/mL	1.01 (0.58–1.78)	0.964
**Male VDBG**	<240 vs. ≥240 µg/mL	0.74 (0.46–1.20)	0.221
**Male Albumin**	<4.5 vs. ≥4.5 g/dL	0.88 (0.44–1.78)	0.726
**Female Free 25(OH)D**	<8.25 vs. 8.25–12.5 pg/mL	2.21 (1.16–4.23)	0.016
	>12.5 vs. 8.25–12.5 pg/mL	3.52 (1.58–7.84)	0.002
**Male Free 25(OH)D**	<8.25 vs. ≥8.25 pg/mL	0.65 (0.37–1.13)	0.128

* OR: odds ratio; CI: confidence interval; VDBG: vitamin D binding globulin

Supplementary Table S4. Fully Adjusted Single-Model Associations Between Maternal and Paternal Preconception Vitamin D Biomarkers and Odds of Preeclampsia with Inverse Probability Weighting (IPW) Restricted to ≥20 Weeks Gestation in the FAZST Trial

Biomarker	Comparison	Adjusted OR (95% CI)	P-value
**Female Total 25(OH)D**	≤20 vs. 30-49 ng/mL	2.26 (1.13-4.50)	0.020
	21–29 vs. 30-49 ng/mL	1.68 (0.93-3.04)	0.084
	≥50 vs. 30-49 ng/mL	3.55 (1.32-9.56)	0.012
**Male Total 25(OH)D**	≤20 vs. ≥30 ng/mL	0.95 (0.50-1.82)	0.885
	21-29 vs. ≥30 ng/mL	0.82 (0.46-1.48)	0.511
**Female Free 25(OH)D**	<8.25 vs. 8.25-12.5 pg/mL	2.06 (1.14-3.74)	0.017
	>12.5 vs. 8.25-12.5 pg/mL	3.73 (1.73-8.08)	0.001
**Male Free 25(OH)D**	<8.25 vs. ≥8.25 pg/mL	0.76 (0.45-1.27)	0.294
**Female Bioavailable 25(OH)D**	<3.5 vs. 3.5-5.0 ng/mL	2.51 (1.23-5.13)	0.012
	>5.0 vs. 3.5-5.0 ng/mL	4.76 (2.09-10.84)	<0.001
**Male Bioavailable 25(OH)D**	<3.5 vs. ≥3.5 ng/mL	0.71 (0.43-1.17)	0.180
**Female VDBG**	<240 vs. 240-280 µg/mL	1.29 (0.75-2.20)	0.353
	≥280 vs. 240-280 µg/mL	1.20 (0.59-2.48)	0.613
**Male VDBG**	<240 vs. ≥240 µg/mL	0.76 (0.47-1.24)	0.266
**Female Albumin**	<4.5 vs. ≥4.5 g/dL	1.45 (0.83-2.54)	0.190

**Male Albumin**	<4.5 vs. ≥4.5 g/dL	0.88 (0.43–1.81)	0.727
